# ‘Hidden Habitus’: A Qualitative Study of Socio-Ecological Influences on Drinking Practices and Social Identity in Mid-Adolescence

**DOI:** 10.3390/ijerph14060611

**Published:** 2017-06-08

**Authors:** Stephanie Scott, Janet Shucksmith, Rachel Baker, Eileen Kaner

**Affiliations:** 1 Institute of Health and Society, Newcastle University, Newcastle upon Tyne NE2 4AX, UK; eileen.kaner@ncl.ac.uk; 2 Health and Social Care Institute, University of Teesside, Middlesbrough TS1 3BA, UK; janet.shucksmith@gmail.com; 3 Yunus Centre for Social Business and Health, Glasgow Caledonian University, Glasgow G4 0BA, UK; Rachel.Baker@gcu.ac.uk

**Keywords:** adolescents, alcohol, identity, marketing, Bourdieu, qualitative

## Abstract

This study explored mid-adolescents’ views and experiences of socio-ecological influences on their drinking practices in order to help inform the development of interventions to reduce alcohol-related risk. We conducted 31 in-depth interviews with young people aged 13–17 in North East England. Verbatim interview transcripts and field notes were coded systematically and analysed thematically, following the principles of constant comparison. We adopted Bourdieu’s idea of social game-playing and elements of his conceptual toolkit (particularly habitus, capital and field) during analysis. Analysis yielded three intersecting themes: (1) ‘drinking etiquette’: conveying taste and disgust; (2) ‘playing the drinking game’: demonstrating cultural competency; (3) ‘hidden habitus’—the role of alcohol marketing. Our work demonstrates that there is a nexus of influential factors which come together to help shape and reinforce mid-adolescents’ behaviour, norms and values in relation to alcohol consumption. Drinking practices are not just formed by friendships and family traditions, these are also subject to wider cultural shaping including by the alcohol industry which can encourage brand identification, and gear specific products to add ‘distinction’. However young people are not inactive players and they use aspects of capital and social games to help cement their identity and present themselves in particular ways which in turn are influenced by age, gender and social status. Guided by promising work in the tobacco field, interventions which focus on critical awareness of the framing of alcohol products by key stakeholders, such as policymakers, commercial industry and public health professionals, and by wider society may facilitate behaviour change among young people.

## 1. Introduction

Alcohol use is the leading cause of death and disability adjusted life years in both 15–19 year-olds and 20–24 year-olds globally [1]. Whilst underage alcohol use—particularly frequent, high-intensity use—increases the probability of short and long term negative consequences relating to physical health [2,3,4,5,6], it also carries concomitant social risk, ranging from the personal (e.g., regretted or unprotected sexual activity, poor school attendance and attainment) to the societal (e.g., disorderly city centres, productivity losses) [7,8,9,10]. Such alcohol-related consequences appear most clearly within a subset of risky drinkers within a broader trend of declining adolescent alcohol consumption, a phenomenon previously described as ‘more alcohol down fewer throats’ [11,12,13,14]. Whilst reported falls in consumption are to be welcomed, there remains a critical need to develop a richer understanding of young people’s evolving drinking practices in order to inform the development of early interventions to reduce alcohol-related risk linked to those that drink. Exposure to (and engagement with) alcohol marketing has been consistently associated with early initiation of drinking and regular use of alcohol amongst young people, as well as the development of pro-drinking attitudes and social norms [15,16,17]. A recent systematic review identified a majority of evidence that linked industry-driven alcohol marketing (Price, Promotion, Product and Place) to key drinking outcomes (initiation, continuation, frequency and intensity) in young adolescents (aged 9–17 years), with evidence strongest for promotional activity [18]. However, null results or negative associations were also found, and a field of highly variable and inconsistent exposure and outcome measures hampered the ability to conduct data pooling.

Marketing is embedded in a rich milieu of other social and cultural influences on behaviour and it is increasingly recognised that behavioural drivers work together to collectively influence young people’s drinking practices. Whilst there are a number of conceptual lenses that could be drawn upon to make sense of why people act the way they do, one way in which to interpret young people’s drinking practices is by adopting Bourdieu’s theory of practice [19,20], a theoretical framework which rests upon three core concepts. Habitus is an embodied history of shared tastes, habits and dispositions [21] or, put another way, a matrix of ‘generative principles’ which provide a practical logic that an individual draws upon with little active conscious intent [20]. Habitus is flexible, with tiny adaptations made through interactions with others; and cumulative, whereby our reactions in the present are rarely exactly the same, due to our experiences in the past [22]. Field is used to describe a person’s objective position in social, physical and digital space. In other words, field constitutes the ‘arena’ or context in which interactions, transactions and events occur [23]. Fields may be inter-related, in that a person’s position in one field can be advantageous in another; and each has a practical logic (habitus) that a person must master to be comfortable in that social space [23]. This means that if we cross into a field in which we do not immediately ‘fit in’, we may try to adapt and make changes to our habitus. Finally, capital denotes three types of materials or assets, with exchange value, that an individual possesses relative to others: social (constituted by networks), cultural (knowledge of what holds distinction), and economic (accumulation of wealth) [24,25,26]. Symbolic capital is the result of elevation of certain combinations of capital above others once they have been legitimised [27,28]. Taken together this triad generates practices (behaviour). 

Bourdieu also presents the inter-related idea of ‘game-playing’ as a functional metaphorical tool [19,20]. To succeed in a given field requires the ability to ‘fit in’ and achieve a sense of belonging or acceptance by a particular group. To do so, Bourdieu suggests that individuals participate in ‘social games’. Like all games, the ability to play involves acquisition of knowledge, and a commitment to the logic and rules of the game, termed ‘Illusio’ by Bourdieu [20]. For the most part, game-playing is relatively unconscious, as an appropriate habitus predisposes actions to be sensible and logical [29]. Further, the earlier a person learns the rules of the game, the more able they are to act without deliberate intent and without awareness of game-playing. However, participation can involve a level of sacrifice and discomfort, at least until socialized properly into the ‘correct way to play’. Adolescence is a time of rapid physical and emotional development—where identity is still being shaped [30,31]. The act of fitting in amongst preferred peers can therefore take a huge amount of physical and emotional effort and involves the legitimizing and exclusion of certain types of people and/or behaviours. It is not easy to be the one who stands out, particularly during adolescence. This means that image and presentation of self is critical [32]. Game-playing offers people a practical sense of how things should be done, described by Bourdieu as ‘legitimate culture’ [20]. In the context of young people’s drinking practices, it could therefore be assumed that adolescence represents ‘field’ with alcohol consumption just one of the ‘games in play’. Thus, habitus may reflect the lexicon of influences upon young people’s drinking practices; meanwhile capital represents the resource a young person could have to enable or facilitate alcohol consumption, such as parental supply (social capital) or disposable income (economic capital).

Aspects of Bourdieu’s theoretical framework have previously been applied to the study of young people’s alcohol use [33,34]. Most recently, Bourdieu’s concepts of habitus, field and capital have been used to explore the attitudes and beliefs of UK young adults (aged 18–20) around alcohol use, particularly in relation to the role of peers [35]. Study authors demonstrated that friends were integral in drinking experiences, and drinking with friends was equated with fun and enjoyment. Critically, however, wider cultural norms played the predominant role in shaping behaviour, via the internalisation of widely accepted practice and the subsequent externalisation of norms through the habitus. 

Previous work in this area only partially links together the role of alcohol marketing and social game-playing with other influential factors within underage drinking practices. Data from Lunnay et al. was collected in Australia and relates to females only [33], whilst MacArthur et al. did not identify alcohol marketing as an important facet of habitus [35]. Further, the latter also focused on young adults aged 18–20, and there are differing and important intervention considerations between groups under and over the UK ‘legal’ age of purchasing alcohol. Therefore, our paper aims to adopt Bourdieu’s idea of social game-playing and elements of his conceptual toolkit (particularly habitus, capital and field) to enhance understanding of the wider drivers of mid-adolescent (aged 13–17) drinking practices with a view to aiding the development and implementation of effective preventative interventions in the UK.

## 2. Materials and Methods

### 2.1. Sampling and Participant Information

Our approach to data collection, coding and analysis was guided by COREQ (COnsolidated criteria for REporting Qualitative research) [36]. Our full COREQ checklist is provided in the supplementary material. Data were derived from qualitative enquiry situated within a broader research project focused on understanding the influence of the commercial environment on mid-adolescent drinking beliefs and behaviours [18,37,38]. 31 in-depth interviews were conducted with young people aged 13–17 who were current users of alcohol at the time of the study. These interviews were conducted between May 2009 and March 2010 and lasted between 45 and 90 min. Most interviews were conducted on a one-to-one basis, whilst six interviews (involving 12 young people) were carried out in dyads, at the request of participants. During one interview a youth worker was present at the request of the participant. Sampling continued until no new issues or perspectives emerged from the interviews and there were indications of ‘data saturation’, later confirmed during data analysis. To achieve maximum variation in young people’s perspectives, participants were purposively recruited on the basis of age, sex and socio-economic backgrounds, and were recruited from a range of different settings (high schools, vocational/further education colleges, youth centres and youth offending teams in North East England). All participants were white British individuals, reflecting the predominant demography of the study area. A summary of participant characteristics (by age, gender, level of deprivation and interview structure) is presented in Table 1 below. The Index of Multiple Deprivation (IMD) 2015 area-level measure of socio-economic position was used as an ecological proxy for individual socio-economic position. Participants were not asked for postcode data therefore the postcode of the recruiting site was used. IMD scores were subsequently converted to quintiles, where ‘1’ indicates ‘most deprived’.

### 2.2. Data Collection

A semi-structured approach was undertaken, with a topic guide used to inform but not direct the interviews. This topic guide was developed iteratively throughout data collection and covered a range of issues including experiences and reasons for drinking alcohol; social networks, peer groups and drinking contexts; tastes and preferences; awareness of and/or engagement with alcohol products and marketing techniques; access to alcohol and the importance of price; and risk and vulnerabilities. All interviews were conducted by the first author (Stephanie Scott), who at the time of the study was a PhD student (educated to Masters Level) and classed as a young female (aged 24 years). As part of her doctorate, the interviewer received detailed training in qualitative interviewing and analysis. 

All participants were approached face-to-face, no young people approached for interview refused to take part and there were no drop-outs during the interview process. Interviews took place at the recruiting site or a nearby public coffee shop. All young people consented to their interview being audio recorded. Interviews were transcribed verbatim, either by the researcher who conducted the interview (Stephanie Scott) or by a research administrator at the university, with observational field notes maintained in a research diary. Ethical approval for the study was provided by Newcastle University (REF 000125/2009, 27/04/2009). No relationship between the interviewer and participants was established prior to study. All participants received a study information leaflet, which included details about the interviewer’s credentials and reasons for conducting the study. Participants provided written informed consent before taking part and anonymity was assured. 

### 2.3. Data Coding and Analysis

Data collection and analysis took place concurrently, in order to continually re-evaluate emergent findings. Verbatim interview transcripts and field notes were analysed thematically [39], following the principles of constant comparison [40] to enhance internal validity [41]. A computer program (Microsoft Excel, Microsoft Corporation, Redmond, WA, USA) was used to assist with the organisational aspects of data coding and analysis. Transcripts were first coded line by line and then systematically indexed into data tables to generate descriptive themes. Descriptive themes were then compared to identify patterns, similarities and differences in the data in order to generate analytical themes. We adopted Bourdieu’s idea of social game-playing and elements of his conceptual toolkit (particularly habitus, capital and field) during analysis. Coding and analysis were undertaken by the researcher who conducted the interviews (Stephanie Scott), with transcripts reviewed by a second researcher (Janet Shucksmith). To maximise analytic potential, emergent themes were discussed and challenged within the entire study team, a process described by Barbour as pragmatic double coding [42]. Analysis of transcripts yielded three intersecting themes: (1) ‘drinking etiquette’: conveying distinction and disgust; (2) ‘playing the drinking game’: demonstrating cultural competency; (3) ‘hidden habitus’—the role of alcohol marketing. The findings presented below include quotations to provide rich description and faithful accounts of the views and experiences of young people in this study. All data were coded anonymously to ensure that participants were not identifiable from their accounts.

## 3. Results

### 3.1. ‘Drinking Etiquette’: Conveying Distinction and Disgust

Drinking practices were used by all interviewees to demonstrate ‘who they were’ as well as to mark out others as being different to them. Whilst some values and behaviours drew young people in this study together, others pulled them apart, leading to the social authorisation of certain practices but not others, and resulting in the distancing of people and/or associated behaviour. Doing so enabled young drinkers to display a sense of expertise and pride in their own drinking practices, as well as imply lack of taste, and even disgust, in the behaviours of others. Here, two predominant approaches to alcohol use emerged—drinking to establish maturity and drinking to lose control. For some young people in our study, it was not acceptable to be ‘out of control’ and they negotiated the boundary or ‘edge’ of alcohol consumption so as not to become ‘too drunk’, become an embarrassment or miss out on enjoying their evening (*‘drinking to get drunk… that’s not really what I want to do… not your entire life focused on getting drunk or whatever, it’s sort of something that happens rather than something that you set out to do…’*—Female, aged 16). These young people demonstrated ‘distinction in restraint’, taking pride in being able to recognise their limits and control the effects of intoxication (*‘My dad, well, what he does say is I’m quite good with alcohol he says I’m self-regulating now…’*—Male, aged 17). As such, they deliberately chose drinks which were not overly strong and did not result in immediate and obvious drunkenness, a practice referred to in the wider literature as the ‘intoxication tightrope’ [43], ‘controlled intake’ [44], ‘just the right buzz’ [45] or ‘calculated hedonism’ [46]. Moreover, some young people described reining in their alcohol consumption by only spending a certain amount of money, planning ahead for how to get home or by looking after their friends (*‘I tend to bring about £10 with me. If it’s a party then maybe £15… that’s a way of limiting myself to not actually drink as much*…’—Female, aged 17). 

For many young people, drinking practices were used to demonstrate taste, maturity, autonomy from the crowd and prestige. Cheap wine and cider were associated with younger drinkers or individuals from a lower socio-economic status, whereas cocktails, champagne, white wine or expensive brandy and whisky were described as mature or ‘sophisticated’ drinks (*‘it’s a charva* [individuals from a lower socio-economic status] *drink. You can tell when I would go into a shop and buy a bottle of Bellabrusco it’s like ‘oh that’s for the kids’…’*—Female, aged 16). At this stage of life, participants did not often readily reflect on or identity with typical class labels. However, this does not mean it was not of importance in drinking practices. One label they did reflect on was ‘charva’ and there was an awareness that being seen as a charva can have negative connotations, with use of this label evoking disgust and contempt. Nevertheless, others appeared to embrace this identity, meaning that, in certain fields, charvas did fit in and belong to particular social groups, with the ‘rules of the game’ shifting in different contexts. For these young people drinking to lose control held distinction, and there was prestige and status in product strength (and volume consumption) over product style. Strong products were specifically chosen to achieve this goal (*‘The night’s better when you’re not sober so you start the night not sober so it’s better from the start’*—Female, aged 17). Consuming ‘weak’ products was felt to defeat the principal objective of drinking and represented a ‘waste of money’ (*‘it doesn’t get you pissed or nowt* [nothing] *and you just think, well I’ve wasted my money on something what’s not going to get us pissed.’*—Male, aged 15). Thus, this grouping of young people drank primarily to get drunk and it was important for it to be obvious that they were drinking alcohol. Economic capital was critical here and recognising the importance of price or knowing where it is easiest to access alcohol were ‘learned’ drinking behaviours (*‘At a pub and club it’s really fast pace getting the alcohol so they just don’t bother* [with ID]. *So you go when it’s busy and they won’t bother, while* [in] *a shop there’s loads of people looking at you and someone might recognise you’*—Female, aged 17). These young people appeared to be interested in cheap or easily accessible alcohol, and were willing to take advantage of ‘freebies’, discounts or special offers (*‘I don’t go out on weekends, I go out during the week so I go out on Monday and Thursday… everything is cheaper, but if I went out on a Saturday then I’d probably have to buy different types of drinks, because I wouldn’t be able to afford it because they’re double the price’*—Female, aged 17). Further, some interviewees were relatively indiscriminate about the type of alcohol they consumed, especially if others were purchasing alcohol. For example, previously scorned drinks and brands became acceptable if they were a free and convenient source of alcohol (*‘I wouldn’t dare touch that, we wouldn’t drink that… it tastes horrible… Unless someone else bought it… Wouldn’t drink it unless I got it for nowt…Wouldn’t waste me money…’*—Male, aged 14). 

### 3.2. ‘Playing the Drinking Game’: Demonstrating Cultural Competency

For young people in our study, drinking alcohol required them to learn and understand distinct ‘rules of play’ and involved the acquisition, maintenance, development and mobilisation of resources or capital (*‘I guess it’s like drinking etiquette’*—Female, aged 17). Those who chose to remain outside the boundaries of acceptable practice (for example, drinking alone or abstaining without good reason e.g., being the designated driver) risked exclusion (*‘[drinking alone is] lonely, desperate, because I do it socially, I’d never do it just by myself… drink, because it’s nice in an atmosphere with friends’*—Female, aged 17). If their drinking behaviour was deemed unacceptable, young people could find themselves ‘frozen-out’, side-lined and lacking in credibility. These transgressions were often managed within the group (*‘we do tend to emphasise to each other that you should know your limitations so if someone doesn’t know his limits then we would be he’s not really that cool to hang out with… I guess there is a borderline between funny and embarrassing… like the new people who come in sometimes they go over the top of it and then they know that that’s not really the way to go, so they kind of buckle down next time’*—Male, aged 17). Further, the earlier in their drinking ‘career’ that interviewees discussed learning the ‘rules of the game’, the more likely it was that drinking practices became normalised and acceptable. Knowing how to ‘play the game’ demonstrated cultural competency, a vital commodity in adolescence. Thus, some younger interviewees in our study described the process of ‘learning’ how to drink: *‘I didn’t like it at first ‘cos I was sick all over and my ma found out… but as I got used to it I was alright… I just love the buzz of getting drunk now’*—Female, aged 13. Doing so took perseverance and commitment, with some young people describing the need to learn to like the taste of alcohol despite describing the initial taste of alcohol as ‘horrible’ or ‘disgusting’. They employed a number of practical strategies to help manage this, such as experimenting with different drinks and masking the taste of alcohol with more palatable products (*‘I like the orange one ‘cos it tastes like Irn Bru…’*—Male, aged 14), a finding which accords well with other studies of alcohol use in mid-adolescence [47], and which reinforces explanations of the alcohol industry’s introduction of ‘alco-pop’ products to the market [48]. Young people also projected any adverse effects experienced, such as vomiting, onto particular products, and described simply learning not to drink that type of alcohol again. Peers were an integral part of the game—alcohol was used to cement friendships and offered young people a sense of ‘community’. Thus, interviewees discussed sharing values, traditions and rituals with others in their social network, which seemed to denote acceptance and belonging (*‘What we basically do is we go in, we get changed, we meet each other, go on Facebook and talk to each other, we meet each other, we get wor [our] money, we go out, sometimes...not all the time, sometimes have a little drink and we just dance about, talk, tell jokes, take pictures and then go in...we don’t talk to anyone, we don’t give grief to anyone, we don’t cause trouble, we just stay in one drinking place. Like if we don’t drink we just go down the park and we just play in the park’*—Female, aged 13). Thus, the value derived from a night out was extended both retrospectively and prospectively, through sharing stories, high levels of planning, getting ready together and use of social networking sites to validate and re-live experiences, a finding which accords well with other literature [49]. Extending the night in these ways heightened the experience of drinking alcohol for young people (*‘you know when you go on Facebook and that, you gan [go] on the next morning and people’s put ‘oh I had a good night last night but hangover now’…Loads put photos on there’*—Male, aged 15). 

Friendships could also shield interviewees from adverse or unwanted consequences of drinking. Thus, it was expected that friends would protect and support each other. For both genders, this meant looking after friends who were drunk. For girls, it also meant that they would not let each other go home alone or with a stranger. Whilst, for boys, this tended to mean sticking together when coming into contact with the police or other groups of young people (*‘Well even if my pals were getting chinned* [assaulted] *and I had been drinking, like other people had started chinning one of my pals, then I would just gan* [go] *over and I would end up fighting, sticking up for my pals…my pal, the one I gan over to the town with to his da’s* [dad’s], *me and him we’re just like that and he knows I’ve always got his back if someone’s going to hit him. I divvent* [don’t] *like fighting and that but I mean if my pals going to get chinned I divvent want my pals getting chinned, I divvent want them getting touched so I stick up for them’*—Male, aged 15). Further, friendships and social networks offered a ‘micro economy’ to allow young people to effectively pool limited resource. Young people took turns to purchase alcohol or ‘subbed’ each other, pooled money together and ‘pre-loaded’ before going out for the night. Whilst interviewees gave a number of reasons for pooling resources—suggesting that this represented a cheaper and fairer system and thus stopped arguments, that alcohol lasted longer that way, and that it enhanced drinking as a social experience—predominantly there seemed to be a reciprocal understanding i.e., ‘if I pay this time, they’ll pay when they have money next time’, which also helped to cement relationships (*‘we’ll buy wor* [our] *own but then if someone hasn’t got money…like my pal… if he hasn’t got money and that...and I’ve got money and I get drink I’ll give him basically half and stuff ‘cause he knows for a fine fact...well I know for fine fact that I’d get it back when he’s got money and I haven’t’*—Male, aged 15). In addition to peer networks, young people drew on their parents when developing new drinking practices (*‘Dad told me how to make Moscow Mules…’*—Male, aged 15). Young people regularly discussed drinking with their parents, suggesting that their parents encouraged them to drink sensibly, and to try alcohol at an early age, in order to eradicate the mystique around drinking alcohol, and as a ‘least worst’ strategy in comparison to smoking and drug taking, findings which accord well with other studies of parental influence on adolescent alcohol use [50,51]: *‘they basically told me just...what’s out there and they tried to introduce me at a young a...just saying no you can have a sip of my wine or whatever so that...the first time you do it, it wouldn’t be like something new that you just go out and do it loads because you’ve never done it before’*—Male, aged 17. 

### 3.3. ‘Hidden Habitus’—The Role of Alcohol Marketing

Alcohol marketing was a key external factor which shaped young people’s drinking behaviour, built up outcome expectancies and reinforced particular identities (*‘I go with my dad to the rugby a lot... we usually have a pint after the match… You often see adverts for alcohol where there are men drinking in the stadium, it has that whole macho culture’*—Male, aged 17). This quote shows how sponsorship of sporting or cultural activities was a key feature of marketing reach; and interviewees frequently recited advertising campaigns or slogans (*“somebody does something bad and it says ‘have you got your WKD side?’”*—Male, aged 14). Meaning was attached to certain products, brands or consumption patterns which denoted affiliation to a particular social group or rejection of another (*‘whenever anybody says Baileys I always think of The Mighty Boosh. There’s an episode, old Greg, he’s like you’re having Baileys from a shoe. That always makes us laugh. I’ve always wanted to try that.’*—Female, aged 16). Certain drinks were described as ‘macho’ (and at times linked to sport), whilst others such as alcopops were described as a drink for ‘wimps’ or girls, and likened to soft drinks (*‘Some drinks the guys would never go near like WKDs, Apple Sours because it’s quite feminine for them, they’d go for the hard tequila…show off…all about image, because they’d get taken the mick out of if they had something that was colourful…it’s more manly to have a drink in your hand I think than to just take little shots.’*—Female, aged 17). Interviewees also tended to suggest that boys appeared to drink at a higher volume or intensity to girls, and that girls and boys tended to ‘act differently’ (i.e., louder, immature or more confident) when drunk (*‘But I think the lads drink much more…much quicker. By the end of the night we’ve all drank our drink and then like girls they’ve barely drank any ‘cause they just sit there and do nothing. Sit there in shame or something’*—Male, aged 16). Highlighting ‘unacceptable’ behaviour at times led to criticism of their own younger selves; with previous behaviour being portrayed as ‘childish’ in comparison to their current approach (*‘I think the novelty’s worn off…it’s not so much of a…oh let’s get drunk for the sake of going and getting drunk, it’s a lot more like alright I’m going out so I’ll have a drink because it’s nice when I’m going out but I’m not going to do it just for the sake of getting drunk…’*—Male, aged 17). Older young people described themselves to have developed greater tolerance levels towards alcohol, and as now able to drink at an increased volume without obvious effect or intoxication. 

As well as criticising their own younger selves, interview participants felt that the drinking behaviour of ‘younger’ young people was more likely to be influenced by external factors such as marketing (*‘I would say if people are first starting to drink then it* [advertising] *would have more of an effect because they won’t really know what’s out there…so they just see a poster…maybe I’ll have one of those see what it tastes like…’*—Male, aged 17). In this way, alcohol advertising was often described as ‘helpful’ to new or younger drinkers by drawing their attention to products in order to help them experiment with alcohol (*‘I seen one of the adverts off Jack Daniels…I was like ‘oh I wonder if I’d do that if I’m on Jackie D’s’ and I just had to get a bottle of Jackie D’s and nowt happened…I liked the taste of it and that. Same with Southern Comfort. I’ve seen the advert for that and I was like ‘oh’ and decided to have a drink of it’*—Male, aged 17). Thus, to some degree, there was an underlying critical consciousness of alcohol advertisements (‘*I don’t particularly notice them. I’m not really into the real alcohol scene like knowing all your brands and knowing all the sophisticated stuff. I’m not really into that*...’— Male, aged 17), with some interviewees seemingly ‘cynical’ in comparison to other people who were susceptible to such influence (*‘They always talk crap’*—Male, aged 16; *‘No ‘cause you can’t taste the adverts*’—Female, aged 17). Finally, advertising had a role to play in drawing attention to price related promotions and special offers. Many practical decisions about what to drink appeared to be framed by the price of alcohol e.g., sharing alcohol, cheap, high-strength products, buying in bulk, drinking ‘house’ spirits and choosing to go to pubs and clubs on certain nights of the week (*‘I don’t go out on weekends, I go out on a Monday and Thursday then everything is cheaper…if I went out on a Saturday then I’d probably have to buy different types of drinks because I wouldn’t be able to afford it because they’re double the price…’*—Female, aged 17). For example, one 15 year old boy commented on his reaction when he saw a supermarket price promotion, *‘You just ring up your mates and say have you seen this, it’s went [gone] down cheaper’.* When asked if price was critical to his choice, he commented: *‘Aye sometimes. ‘Cause if I haven’t got enough I’ve got to get something else*’; whilst some interviewees also articulated the influence of visual promotions in pubs and clubs, often related to price (*‘if you were in a club and you had been drinking and there’s like advertisement that’s saying it’s cheap or something, I’d probably go for that…’*—Female, aged 17). 

## 4. Discussion

Different forms of alcohol consumption were used to help construct identity, cement young people’s place within their peer group and mark out others as being ‘different’ to them. Whilst some young people reported that they drank to demonstrate maturity, others felt that they drank to lose control. These findings are supported by other studies conducted with young people, especially with regard to: established and embedded drinking norms or dispositions [52], the symbolic value attached to drinking behaviour [53], the importance of ‘belonging’ to a particular peer group [35,54], the use of alcohol to help construct particular forms of social identity [55], and the importance of the social and cultural environment in shaping drinking practices [33,56]. In particular, the distancing or ridiculing of those who transgressed the boundaries of acceptable behaviour was a recurring theme and this has been reported in other work, particularly in the context of adolescent food and clothing choices [57,58]. Alcohol consumption was used by the young people in this study—like fashion or a hairstyle—as a shortcut to creating or conferring identity, with their choice of drink serving to demonstrate ‘who they are’. Further, the ‘othering’ of different class, gender and age groups led to numerous examples of ‘banter’ or derogatory language, such as ‘wimp’ and ‘charva’, reflecting previous work with a focus on consumption practices and ‘youth underclass’ [59,60]. This finding also accords well with recent work conducted with 14–16 year-olds boys exploring race and what it means to be a white working class male in South London [61]; as well as research conducted with working-class men in Scotland demonstrating a gender and sexual identity hierarchy within drinking values and practices [62]. Here, adult interviewees articulated that there was a ‘proper’ way of drinking, with women and gay men having subordinate status, reinforcing hegemonic masculinity [63] or ‘hyper-gender’ roles [64,65]. In our study, just one interviewee identified as gay. Research has highlighted that lesbian, gay, bisexual and transgender (LGBT) youth use alcohol and drugs to a greater degree than heterosexual people [66]. However, this aspect of use is under-explored and less understood, thus further work with adolescents exploring the role of sexual identity in alcohol consumption is warranted. It has been questioned whether social class remains a useful construct to draw upon [67]. For interviewees in this study, even at a young age, social class was frequently used to make value judgements about others, coded in the way others talked, the things they wore, and in the way that they drank alcohol. Class differences (even where not explicitly framed as such by adolescents in this study) operated as useful cultural capital to help individuals to fit in, reproducing ‘classed exclusion’. However, in this study, exclusionary tactics, guided by stigma, taste and disgust, were used by both affluent and deprived young people, suggesting that both groups can be thought to possess ‘legitimate culture’ within their own particular social space, and according to their habitus.

What this and other studies indicate is that youth drinking can only be fully understood within a wider social and societal context. Whilst industry-driven marketing is an important aspect of the external environment for young people, it seems to be firmly embedded alongside other key influences such as cultural and geographical traditions, peer groups and family-based drinking practices. In our study, we saw an interplay between aspects of the internal and external world which helped to build early social identity, drive learning and shape what individuals and others around them do. Further, others have suggested that young people inhabit an ‘intoxigenic’ environment where social, physical, cultural and commercial influences shape youth drinking but are not always consciously recognised as doing so [68]. Such influences have also been described as ‘affordances’, where properties in the environment have functional significance for individuals and provide opportunities or settings for certain behaviours [69]. Our work suggests that young people are not inactive players in this social world but rather that they actively seek out experiences which help build specific identities that draw them together with some groups (e.g., desired peers) and distinguishes or ‘sets them apart’ from others. Marketing appears to reinforce aspects of the surrounding social ecology, by encouraging a link between alcohol and aspects of culture, identity and personal reward [21]. Our work does not, and cannot, ascribe intentionality on the part of the alcohol industry with regard to young people. However, marketing relationships specifically built up with adults [70] seem to also impact on young drinkers and become part of the way they construct their early identity, well before the age at which they can legally purchase alcohol [38]. Gender, class and sexuality are just some of the aspects of identity that marketers can use to help sell more of their product in the shorter and longer term. An analysis of internal alcohol industry advertising documents demonstrated projected brand values of masculinity and bravado as well as other sexual stereotypes [71]. Nevertheless, research relating to alcohol marketing continues to focus heavily upon the effect of traditional advertising exposure [72]. However, this study adds to a growing body of work indicating that future studies of alcohol marketing need to consider how an engagement rather than exposure model of commercial influence may be key to understanding how industry contribute to shaping and nurturing drinking behaviours [73]. Indeed the boundaries between commercial marketing and user-generated content are increasingly blurred; trends such as real-world tie-ins, interactive games and competitions reflect a dialogue and enduring relationship with consumers [74]. 

An engagement model of marketing extends to nightlife activity, often referred to as the ‘P’ for ‘Place’ in the marketing lexicon. Historically, differences in the two core ‘traditions’ of youth research—youth transitions and youth cultural analyses—have resulted in a fragmented, dichotomous youth studies field. However, recently, Hollands has reflected on points of convergence, one of which is around studies which have begun looking at the structuring of nightlife activity and what this can tell us about where and how social inequalities and distinctions are displayed and acted out by young people [75]. In particular, he notes the growing number of pubs and clubs owned by national/multinational companies, resulting in corporate branding and theming, standardised, predictable nightlife experiences and the policing of entry through ‘style pickers’ on the doors and gentrification symbols, thus creating a socially differentiated market [76]. Hollands concludes: “Contrary to the notion that nocturnal youth activity is simply a matter of personal taste and is freely chosen, Chatterton and Hollands (2003:8) argue, paraphrasing Marx: ‘youth make their own nightlife but not under conditions of their own making’” (2015:77), resulting in the structured inequalities in consumption we discuss in our study, verbal class-based conflict and distinctions, and involving what Bourdieu [19], and others [77] have referred to as acts of ‘symbolic’ or ‘structural’ violence.

This study has some limitations which warrant discussion. Our research was conducted with one sample of young people aged 13–17 within one geographical area of the UK, which is a part of England with a strong industrial past and a traditionally heavy drinking culture, reflected in some traditional gender views which may not be found in other cosmopolitan areas. Whilst interviews were conducted in 2010, our analysis and subsequent findings resonate with other work in the field of adolescent drinking. All participants in this study were current drinkers, since we wished to understand the influence of contextual factors on drinking behaviour rather than the decision to drink or not in the first place. Nevertheless, the absence of views from individuals who chose not to drink alcohol is a limitation of our work on shaping influences, i.e., what makes the difference between youth living ostensibly in the same external environment who do and do not drink? Bourdieu suggests that, in order to fit in, one must be able to keep up with changes in the field. Further, that falling behind may induce ‘hysteresis’, defined as a mismatch between habitus and field [19]. Such mismatches can accommodate and allow for societal change. A recent trend of declining adolescent alcohol consumption may be indicative of such societal change, and it may be of interest in future work to explore non-drinking using this theoretical lens. A small proportion of the interviews were conducted with dyads (two young people together) and this approach helped reduce the possible power differential between young people and the adult interviewer. Most dyadic interviews were conducted with younger interviewees in predominantly female same-sex pairs (see Table 1). We considered whether dyadic interviews could lead to socially desirable responses, or boasting about drinking behaviour in front of peers. Thus, in order to minimise socially desirable responses, young people were probed about their responses throughout interviews, and data collected during dyadic interviews was compared to that which was collected during one-to-one encounters, in order to look for signs of hubris or exaggerated claims about drinking. Finally, as participants in this study were predominantly recruited from transient settings and could not be re-contacted for a number of reasons (e.g., no longer attending targeted youth groups, moving away to university), interviewees were not provided with a verbatim transcript of their interview for review. Nevertheless, whilst so-called ‘member checks’ are widely employed by researchers across numerous fields, the advantages of its use in relation to verifying accuracy have been described as relatively small [78], and unlikely to be relevant to research focused on theory development [79].

## 5. Conclusions

This study provides useful insights for future alcohol prevention policy and practice. All interviewees were below the UK legal age of being able to buy alcohol. Nevertheless, they often described themselves as ‘well practised’ in the use of alcohol and they often described ‘learning’ how to be so. In some instances parents were clearly initiating or ‘instructing’ them, perhaps in an attempt to mitigate the various physical and psycho-social risks to these young people whilst growing up, particularly in public drinking environments. The finding that risky or heavy drinking or loss of control due to alcohol can have a ‘cultural logic’ to specific groups of young people is important. This has been recognised by other authors, particularly in relation to young adults, gender and alcohol use [70]. Consequently, what may seem irrational in public health or health policy terms, could be completely rational to individuals if it supports membership of specific peer groups. The data in this study suggest young people achieved a sense of specific personal identity linked to different forms of drinking, highlighting heterogeneity in attitudes and practices, and indicating that simple health education to counter the negative influence of the alcohol environment on young people may not achieve a substantial impact. Only a relatively small number of campaigns or intervention programmes have reported positive outcomes in this area [80]. Previous work applying Bourdieu’s theory of practice suggests that population-level interventions that regulate alcohol consumption at a higher system level, and thus disrupt the field, are likely to facilitate behaviour change among young people by driving a response in habitus [35]. However, such high-level structural change requires political will, if it is to be implemented, which is currently not found in England and Wales, though is perhaps in Scotland. Data from this study and previous studies from the tobacco field, such as the ‘Florida Truth Campaign’, suggest that intervention approaches which capitalise on ‘cynicism’ regarding stakeholder messages and which focus on critical awareness of how alcohol products and use may be framed could offer a promising alternative to individualized health-focused behaviour change models in lieu of population-level change [81,82]. Whilst adverse health and social consequences of early alcohol use remain important, it is the more proximal physiological and emotional outcomes, such as anxieties surrounding weight and self-image, which are key to adolescents a long time before chronic conditions such as liver disease become an issue. Time perspective describes the extent to which people’s value of a reward, such as better health, decreases with delay of its receipt [83,84]. In other words, for adolescents, the positives of alcohol use in the short term outweighs long term negative outcomes. This becomes particularly pertinent if we think of alcohol consumption, ‘going out’ and engagement with urban nightlife as less of a youthful ‘rite of passage’ and more of a permanent post-adolescent ‘socialising ritual’ [85]. Further, it is increasingly recognised that adolescent health and lifestyle behaviours tend to cluster, rather than be experienced in isolation, leading to a growing emphasis on multiple health behaviours [86]. Clearly there remains a need for more work in this area including a focus on both young people themselves and the nexus of wider influential factors, including some which are commercial and some formed from friendships and family traditions, which come together to help shape and reinforce adolescent behaviour, norms and values in relation to alcohol consumption.

## Figures and Tables

**Table 1:** Characteristics of interviewees.

Demographics		*N* (31)	%	*N* of Interview Dyads (12)
Age	13	1	3	0
	14	6	19	6
	15	8	26	4
	16	7	23	0
	17 ^1^	9	29	2
Gender	Female	16	52	9
	Male	15	48	3
IMD quintile	1	20	64	
	2	4	13	
	3	3	10	
	4	4	13	
	5	0	0	

^1^ One participant turned 18 between recruitment and interview.

## Supplementary Materials

The following are available online at www.mdpi.com/1660-4601/14/6/611/s1, COREQ (Consolidated criteria for Reporting Qualitative research) Checklist.

## References

[1] Mokdad A.H., Forouzanfar M.H., Daoud F., Mokdad A.A., El Bcheraoui C., Moradi-Lakeh M., Kyu H.H., Barber R.M., Wagner J., Cercy K. (2016). Global burden of diseases, injuries, and risk factors for young people’s health during 1990–2013: A systematic analysis for the global burden of disease study 2013. Lancet.

[2] Degenhardt L., O’Loughlin C., Swift W., Romaniuk H., Carlin J., Coffey C., Hall W., Patton G. (2013). The persistence of adolescent binge drinking into adulthood: Findings from a 15-year prospective cohort study. BMJ Open.

[3] Rossow I., Kuntsche E. (2013). Early onset of drinking and risk of heavy drinking in young adulthood. A 13-year prospective study. Alcohol Clin. Exp. Res..

[4] Bagnardi V., Rota M., Botteri E., Tramacere I., Islami F., Fedirko V., Scotti L., Jenab M., Turati F., Pasquali E. (2015). Alcohol consumption and site-specific cancer risk: A comprehensive dose-response meta-analysis. Br. J. Cancer.

[5] Holmes M.V., Dale C.E., Zuccolo L., Silverwood R.J., Guo Y., Ye Z., Prieto-Merino D., Dehghan A., Trompet S., Wong A. (2014). Association between alcohol and cardiovascular disease: Mendelian randomisation analysis based on individual participant data. BMJ.

[6] Secretan B., Straif K., Baan R., Grosse Y., El Ghissassi F., Bouvard V., Benbrahim-Tallaa L., Guha N., Freeman C., Galichet L. (2009). A review of human carcinogens—Part E: Tobacco, areca nut, alcohol, coal smoke, and salted fish. Lancet Oncol..

[7] Witt E. (2010). Research on alcohol and adolescent brain development: Opportunities and future directions. Alcohol Alcohol..

[8] Bacharach S.B., Bamberger P., Biron M. (2010). Alcohol consumption and workplace absenteeism: The moderating effect of social support. J. Appl. Psychol..

[9] Hughes K., Bellis M.A., Leckenby N., Quigg Z., Hardcastle K., Sharples O., Llewellyn D.J. (2014). Does legislation to prevent alcohol sales to drunk individuals work? Measuring the propensity for night-time sales to drunks in a UK city. J. Epidemiol. Community Health.

[10] MacLean S., Moore D. (2014). ‘Hyped up’: Assemblages of alcohol, excitement and violence for outer-suburban young adults in the inner-city at night. Int. J. Drug Policy.

[11] Ogeil R.P., Gao C.X., Rehm J., Gmel G., Lloyd B. (2016). Temporal changes in alcohol-related mortality and morbidity in australia. Addiction.

[12] Fuller E. (2015). Smoking, Drinking and Drug Use Among Young People in England in 2014.

[13] Pennay A., Livingston M., MacLean S. (2015). Young people are drinking less: It is time to find out why. Drug Alcohol Rev..

[14] Measham F. (2008). The turning tides of intoxication: Young people’s drinking in britain in the 2000s. Health Educ..

[15] Anderson P., De Bruijn A., Angus K., Gordon R., Hastings G. (2009). Impact of alcohol advertising and media exposure on adolescent alcohol use: A systematic review of longitudinal studies. Alcohol Alcohol..

[16] Jernigan D., Noel J., Landon J., Thornton N., Lobstein T. (2016). Alcohol marketing and youth alcohol consumption: A systematic review of longitudinal studies published since 2008. Addiction.

[17] Stautz K., Brown K.G., King S.E., Shemilt I., Marteau T.M. (2016). Immediate effects of alcohol marketing communications and media portrayals on consumption and cognition: A systematic review and meta-analysis of experimental studies. BMC Public Health.

[18] Scott S., Muirhead C., Shucksmith J., Tyrrell R., Kaner E. (2017). Does industry-driven alcohol marketing influence adolescent drinking behaviour? A systematic review. Alcohol Alcohol..

[19] Bourdieu P. (1984). Distinction.

[20] Bourdieu P. (1990). The Logic of Practice.

[21] Brierley-Jones L.K., Ling J., Wilson G.B., Smith K.E., Haighton C.A., Kaner E.F., Crosland A. (2014). Patterns of middle class alcohol use: Habituses of ‘home’ and ‘traditional’ drinking. Sociol. Health Illn..

[22] Addison M. (2016). Social Games and Identity in the Higher Education Workplace. Playing with Gender, Class and Emotion.

[23] Thomson P., Grenfell M. (2008). Field. Pierre Bourdieu: Key Concepts.

[24] Christensen V.T., Carpiano R.M. (2014). Social class differences in bmi among danish women: Applying cockerham’s health lifestyles approach and bourdieu’s theory of lifestyle. Soc. Sci. Med..

[25] Browne-Yung K., Ziersch A., Baum F. (2013). ‘Faking til you make it’: Social capital accumulation of individuals on low incomes living in contrasting socio-economic neighbourhoods and its implications for health and wellbeing. Soc. Sci. Med..

[26] Demant J., Jarvinen M. (2011). Social capital as norms and resources: Focus groups discussing alcohol. Addict. Res. Theory.

[27] Lawler S. (1999). ‘Getting out and getting away’: Women’s narratives of class mobility. Fem. Rev..

[28] Skeggs B. (1997). Formations of Class and Gender: Becoming Respectable.

[29] Johnson R., Bourdieu P. (1993). Editors introduction: Pierre bourdieu on art, literature and culture. The Field of Cultural Production.

[30] Steinberg L. (2005). Cognitive and affective development in adolescence. Trends Cogn. Sci..

[31] Somerville L.H., Jones R.M., Casey B.J. (2010). A time of change: Behavioral and neural correlates of adolescent sensitivity to appetitive and aversive environmental cues. Brain Cogn..

[32] France A. (2007). Understanding Youth in Late Modernity.

[33] Lunnay B., Ward P., Borlagdan J. (2011). The practise and practice of bourdieu: The application of social theory to youth alcohol research. Int. J. Drug Policy.

[34] Jarvinen M., Gundelach P. (2007). Teenage drinking, symbolic capital and distinction. J. Youth Stud..

[35] MacArthur G., Jacob N., Pound P., Hickman M., Campbell R. (2016). Among friends: A qualitative exploration of the role of peers in young people’s alcohol use using bourdieu’s concepts of habitus, field and capital. Sociol. Health Illn..

[36] Tong A., Sainsbury P., Craig J.C. (2007). Consolidated criteria for reporting qualitative research (coreq): A 32-item checklist for interviews and focus groups. Int. J. Qual. Health Care.

[37] O’Neil S. (2012). Exploring Industry Driven Marketing Influences on Young People Who Drink Alcohol.

[38] Scott S., Baker R., Shucksmith J., Kaner E. (2014). Autonomy, special offers and routines: A q methodological study of industry-driven marketing influences on young people’s drinking behaviour. Addiction.

[39] Silverman D. (2005). Doing Qualitative Research.

[40] Charmaz K. (2006). Constructing Grounded Theory: A Practical Guide through Qualitative Analysis.

[41] Barbour R.S. (2001). Checklists for improving rigour in qualitative research: The case of the tail wagging the dog?. Br. Med. J..

[42] Barbour R.S. (2003). The newfound credibility of qualitative research? Tales of technical essentialism and co-option. Qual. Health Res..

[43] Percy A., Wilson J., McCartan C., McCrystal P. (2011). Teenage Drinking Cultures.

[44] Ander B., Abrahamsson A., Bergnehr D. (2017). ‘It is ok to be drunk, but not too drunk’: Party socialising, drinking ideals, and learning trajectories in swedish adolescent discourse on alcohol use. J. Youth Stud..

[45] Aresi G., Pedersen E.R. (2016). ‘That right level of intoxication’: A grounded theory study on young adults’ drinking in nightlife settings. J. Youth Stud..

[46] Measham F., Brain K. (2005). ‘Binge’ drinking, british alcohol policy and the new culture of intoxication. Crim. Media Cult..

[47] Acier D., Kindelberger C., Chevalier C., Guibert E. (2014). “I always stop before i get sick”: A qualitative study on french adolescents alcohol use. J. Subst. Use.

[48] Measham F. (2006). The new policy mix: Alcohol, harm minimisation and determined drunkenness in contemporary society. Int. J. Drug Policy.

[49] Niland P., Lyons A.C., Goodwin I., Hutton F. (2014). ‘See it doesn’t look pretty does it?’: Young adults’ airbrushed drinking practices on facebook. Psychol. Health.

[50] Jones S.C., Andrews K., Berry N. (2016). Lost in translation: A focus group study of parents’ and adolescents’ interpretations of underage drinking and parental supply. BMC Public Health.

[51] Jacob N., MacArthur G.J., Hickman M., Campbell R. (2015). A qualitative investigation of the role of the family in structuring young people’s alcohol use. Eur J. Public Health.

[52] Advocat J., Lindsay J. (2013). To drink or not to drink? Young australians negotiating the social imperative to drink to intoxication. J. Sociol..

[53] Hackley C., Bengry-Howell A., Griffin C., Mistral W., Szmigin I., Hackley R.A. (2012). Young adults and ‘binge ’ drinking: A bakhtinian analysis. J. Mark. Manag..

[54] Johnson P. (2013). ‘You think you’re a rebel on a big bottle’: Teenage drinking, peers and performance authenticity. J. Youth Stud..

[55] Tutenges S., Rod M.H. (2009). ‘We got incredibly drunk....It was damned fun’: Drinking stories among danish youth. J. Youth Stud..

[56] Townshend T. (2013). Youth, alcohol and place-based leisure behaviours: A study of two locations in England. Soc. Sci. Med..

[57] Stead M., McDermott L., MacKintosh A.M., Adamson A. (2011). Why healthy eating is bad for young people’s health: Identity, belonging and food. Soc. Sci. Med..

[58] Wooten D.B. (2006). From labeling possessions to possessing labels: Ridicule and socialization among adolescents. J. Consum. Res..

[59] Hayward K., Hobbs D. (2007). Beyond the binge in ‘booze britain’: Market-led liminalization and the spectacle of binge drinking. Br. J. Sociol..

[60] Hayward K., Yar M. (2006). The ‘chav’ phenomenon: Consumption, media and the construction of a new underclass. Crim. Media Cult..

[61] Stahl G. (2017). The practice of ‘othering’ in reaffirming white working-class boys’ conceptions of normative identities. J. Youth Stud..

[62] Emslie C., Lennox J.C., Ireland L. (2015). The Social Context of Lgbt People’s Drinking in Scotland.

[63] Connell R.W., Messerschmidt J.W. (2005). Hegemonic masculinity. Rethinking the concept. Gend. Soc..

[64] Anoop N. (2006). Displaced masculinities: Chavs, youth and class in the post-industrial city. Sociology.

[65] Lyons A.C., Willott S.A. (2008). Alcohol consumption, gender identities and women’s changing social positions. Sex Roles.

[66] Kelly J., Davis C., Schlesinger C. (2015). Substance use by same sex attracted young people: Prevalence, perceptions and homophobia. Drug Alcohol Rev..

[67] Urry J. (2000). Sociology beyond Societies: Mobilities for the Twenty-First Century.

[68] McCreanor T., Lyons A., Griffin C., Goodwin I., Barnes H.M., Hutton F. (2013). Youth drinking cultures, social networking and alcohol marketing: Implications for public health. Crit. Public Health.

[69] Heft H., Ward Thompson C., Aspinal P., Bell S. (2010). Affordances and the perception of the landscape: An enquiry into environmental perception and aesthetics. Innovative Approaches to Researching Landscape and Health.

[70] Griffin C., Szmigin I., Bengry-Howell A., Hackley C., Mistral W. (2013). Inhabiting the contradictions: Hypersexual femininity and the culture of intoxication among young women in the uk. Fem. Psychol..

[71] Hastings G. (2010). ‘They’ll Drink Bucket Loads of the Stuff’: An Analysis of Internal Industry Advertising Documents.

[72] Lobstein T., Landon J., Thornton N., Jernigan D. (2017). The commercial use of digital media to market alcohol products: A narrative review. Addiction.

[73] Carah N., Meurk C. (2017). We need a media platform perspective on alcohol marketing: A reply to lobstein et al.. Addiction.

[74] Nicholls J. (2012). Everyday, everywhere: Alcohol marketing and social media—Current trends. Alcohol Alcohol..

[75] Hollands R., Woodman D., Bennett A. (2015). Waiting for the weekend: Nightlife studies and the convergence of youth transition and youth cultural analyses. Youth Cultures, Transitions, and Generations: Bridging the Gap in Youth Research.

[76] Laam H. (2011). Dilemmas of the nightlife fix. Urban Stud..

[77] Lewis S., Russell A. (2013). Young smokers’ narratives: Public health, disadvantage and structural violence. Sociol. Health Illn..

[78] Hagens V., Dobrow M.J., Chafe R. (2009). Interviewee transcript review: Assessing the impact on qualitative research. BMC Med. Res. Methodol..

[79] Thomas D.R. (2017). Feedback from research participants: Are member checks useful in qualitative research?. Qual. Res. Psychol..

[80] Foxcroft D.R., Tsertsvadze A. (2012). Universal alcohol misuse prevention programmes for children and adolescents: Cochranec systematic reviews. Perspect. Public Health.

[81] Taylor J., Taylor A., Lewis S., McNeill A., Britton J., Jones L.L., Bauld L., Parrott S., Wu Q., Szatkowski L. (2016). A qualitative evaluation of a novel intervention using insight into tobacco industry tactics to prevent the uptake of smoking in school-aged children. BMC Public Health.

[82] Sly D.F., Heald G.R., Ray S. (2001). The florida “truth” anti-tobacco media evaluation: Design, first year results, and implications for planning future state media evaluations. Tob. Control.

[83] Barlow P., Reeves A., McKee M., Galea G., Stuckler D. (2016). Unhealthy diets, obesity and time discounting: A systematic literature review and network analysis. Obes. Rev..

[84] Adams J., Nettle D. (2009). Time perspective, personality and smoking, body mass, and physical activity: An empirical study. Br. J. Health Psychol..

[85] Chatterton P., Hollands R. (2001). Changing Our ‘Toon’. Youth, Nightlife and Urban Change in Newcastle.

[86] Kipping R.R., Smith M., Heron J., Hickman M., Campbell R. (2014). Multiple risk behaviour in adolescence and socio-economic status: Findings from a UK birth cohort. Eur. J. Public Health.

